# Supplementary material for: Geographical and social isolation drive the evolution of Austronesian languages
Source: PLoS One. 2020 Dec 1;15(12):e0243171. doi: 10.1371/journal.pone.0243171 (PMC7707576; doi:10.1371/journal.pone.0243171)
Supplement: S1 Fig — It is composed of all the languages included in Gray et al. [39] for which the Pulotu [69] database had an entry. (DOCX) [file pone.0243171.s001.docx]

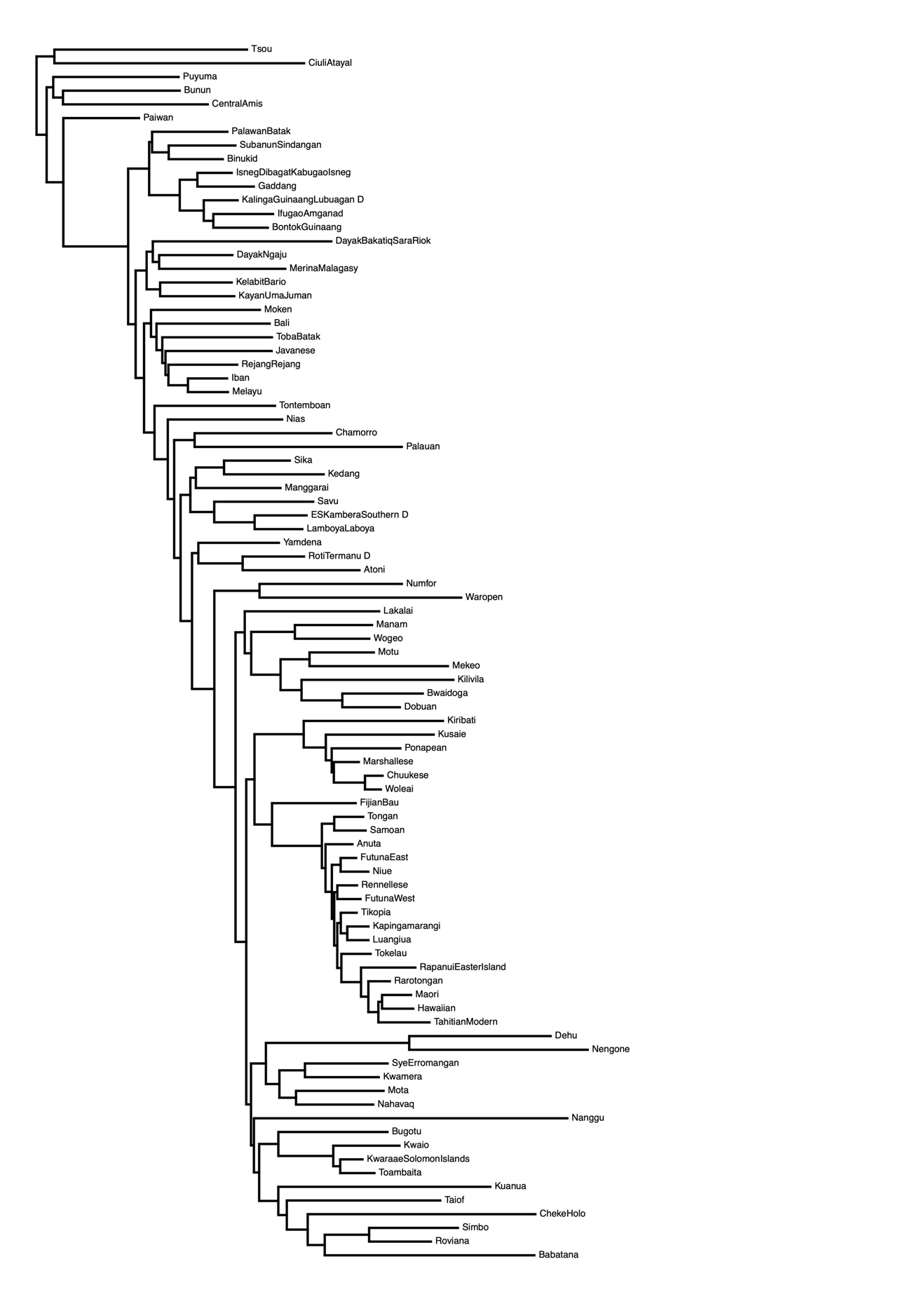
Fig. S1.

Phylogenetic tree used to extract the sister pairs used in our analyses. It is composed of all the languages included in Gray et al.^39^ for which the Pulotu^69^ database had an entry.
