# Supplementary material for: Geographical and social isolation drive the evolution of Austronesian languages
Source: PLoS One. 2020 Dec 1;15(12):e0243171. doi: 10.1371/journal.pone.0243171 (PMC7707576; doi:10.1371/journal.pone.0243171)
Supplement: S2 Fig — Numbers within the cells are Pearson’s correlation coefficients. Blue cells are statistically significant (p < 0.05), with bluer colors as p approaches zero; white cells are borderline statistically significant; red and grey cells are not statistically significant. (DOCX) [file pone.0243171.s002.docx]

Fig. S2.


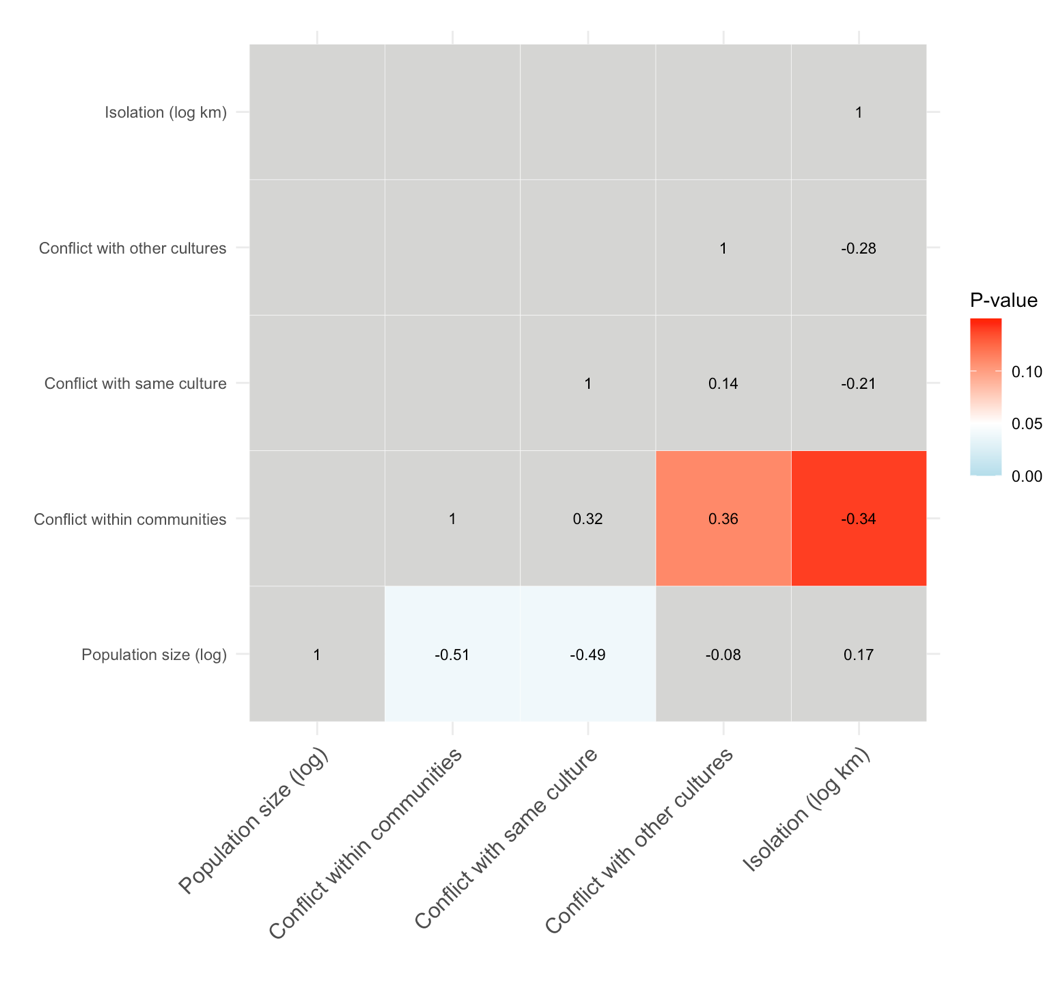


Bivariate correlations between all the predictor variables included in the full model. Numbers within the cells are Pearson’s correlation coefficients. Blue cells are statistically significant (*p* < *0.05),* with bluer colors as *p* approaches zero; white cells are borderline statistically significant; red and grey cells are not statistically significant.
