# Supplementary material for: Geographical and social isolation drive the evolution of Austronesian languages
Source: PLoS One. 2020 Dec 1;15(12):e0243171. doi: 10.1371/journal.pone.0243171 (PMC7707576; doi:10.1371/journal.pone.0243171)
Supplement: S1 Table — The branch length indicates the number of years since the two languages diverged from a common ancestor, as obtained from RateCounter [11]. (DOCX) [file pone.0243171.s003.docx]

Table S1. Sister pairs included in our analysis. The branch length indicates the number of years since the two languages diverged from a common ancestor, as obtained from *RateCounter*^11^.

| **Pair number** | **Branch length** |  | **ISO 16^th^ ED** | **Language name** | **Austronesian subgroup** |
| --- | --- | --- | --- | --- | --- |
|  |  |  | syb | Subanen Central | Philippine |
| 1 | 420 |  | bkd | Binukid | Philippine |
|  |  |  | gad | Gaddang | Philippine |
| 2 | 139.7 |  | isd | Isnag | Philippine |
|  |  |  | bnc | Bontoc Central | Philippine |
| 3 | 284.4 |  | ifa | Ifugao Amganad | Philippine |
|  |  |  | plt | Malagasy Plateau | Western Malayo-Polynesian |
| 4 | 2514.1 |  | nij | Ngaju | Western Malayo-Polynesian |
|  |  |  | kzi | Kelabit | Western Malayo-Polynesian |
| 5 | 2349.9 |  | ree | Kayan Rejang | Western Malayo-Polynesian |
|  |  |  | iba | Iban | Western Malayo-Polynesian |
| 6 | 541.2 |  | zlm | Malay | Western Malayo-Polynesian |
|  |  |  | pau | Palauan | Western Malayo-Polynesian |
| 7 | 3547.7 |  | cha | Chamorro | Western Malayo-Polynesian |
|  |  |  | ksx | Kedang | Central Malayo-Polynesian |
| 8 | 2610.5 |  | ski | Sika | Central Malayo-Polynesian |
|  |  |  | lmy | Lamboya | Central Malayo-Polynesian |
| 9 | 1872.3 |  | xbr | Kambera | Central Malayo-Polynesian |
|  |  |  | aoz | Uab Meto | Central Malayo-Polynesian |
| 10 | 1224.4 |  | twu | Termanu | Central Malayo-Polynesian |
|  |  |  | wrp | Waropen | South Halmahera/ West New Guinea |
| 11 | 932.6 |  | bhw | Biak | South Halmahera/ West New Guinea |
|  |  |  | mva | Manam | - |
| 12 | 171.1 |  | woc | Wogeo | - |
|  |  |  | mek | Mekeo | Papuan Tip |
| 13 | 992.1 |  | meu | Motu | Papuan Tip |
|  |  |  | bwd | Bwaidoka | Papuan Tip |
| 14 | 1066.3 |  | dob | Dobu | Papuan Tip |
|  |  |  | chk | Chuukese | Micronesian |
| 15 | 851.5 |  | woe | Woleaian | Micronesian |
|  |  |  | smo | Samoan | Central Pacific |
| 16 | 1769.1 |  | ton | Tongan | Central Pacific |
|  |  |  | niu | Niue | Polynesian |
| 17 | 1376.5 |  | fud | Futuna East | Polynesian |
|  |  |  | fut | Futuna Aniwa | Polynesian |
| 18 | 1570.4 |  | mnv | Rennell Bellona | Polynesian |
|  |  |  | kpg | Kapingamarangi | Polynesian |
| 19 | 227.1 |  | ojv | Ontong Java | Polynesian |
|  |  |  | mri | Maori | Polynesian |
| 20 | 316.8 |  | haw | Hawaiian | Polynesian |
|  |  |  | nen | Nengone | Southern Oceanic |
| 21 | 1722.1 |  | dhv | Dehu | Southern Oceanic |
|  |  |  | erg | Sie | Southern Oceanic |
| 22 | 1843.3 |  | tnk | Kwamera | Southern Oceanic |
|  |  |  | mtt | Mota | Southern Oceanic |
| 23 | 1029.2 |  | sns | South West Bay | Southern Oceanic |
|  |  |  | mlu | Toabaita | South-East Solomonic |
| 24 | 197.9 |  | kwf | Kwaraae | South-East Solomonic |
|  |  |  | sbb | Simbo | Meso-Melanesian |
| 25 | 631.7 |  | rug | Roviana | Meso-Melanesian |
