# Supplementary material for: Geographical and social isolation drive the evolution of Austronesian languages
Source: PLoS One. 2020 Dec 1;15(12):e0243171. doi: 10.1371/journal.pone.0243171 (PMC7707576; doi:10.1371/journal.pone.0243171)
Supplement: S2 Table — (DOCX) [file pone.0243171.s004.docx]

Table S2. Model comparisons for word gains models.

| **Word gains models** | **WAIC** | **SE** | **weight** |
| --- | --- | --- | --- |
| Population size (log) + Isolation + Conflict | 227.18 | 46.88 | 1.00 |
| Population size + Conflict | 246.99 | 56.56 | 0.00 |
| Conflict within communities | 365.53 | 84.66 | 0.00 |
| Conflict between cultures | 379.76 | 88.02 | 0.00 |
| Conflict within culture | 389.86 | 92.09 | 0.00 |
| Population size (log) | 496.93 | 117.62 | 0.00 |
| Population size (log) + Isolation | 502.37 | 110.54 | 0.00 |
| Intercept-only | 544.48 | 102.32 | 0.00 |
| Isolation | 571.25 | 111.12 | 0.00 |
