# Supplementary material for: Geographical and social isolation drive the evolution of Austronesian languages
Source: PLoS One. 2020 Dec 1;15(12):e0243171. doi: 10.1371/journal.pone.0243171 (PMC7707576; doi:10.1371/journal.pone.0243171)
Supplement: S3 Table — (DOCX) [file pone.0243171.s005.docx]

**Table S3.** Model comparisons for word loss models

| **Word losses models** | **WAIC** | **SE** | **weight** |
| --- | --- | --- | --- |
| Population size (log) + Isolation + Conflict | 206.92 | 47.45 | 1.00 |
| Population size + Conflict | 301.47 | 63.37 | 0.00 |
| Conflict within culture | 372.49 | 80.35 | 0.00 |
| Conflict within communities | 380.13 | 62.25 | 0.00 |
| Conflict between cultures | 389.04 | 68.42 | 0.00 |
| Population size (log) + Isolation | 533.72 | 150.75 | 0.00 |
| Population size (log) | 572.5 | 179.35 | 0.00 |
| Intercept-only | 615.34 | 194.91 | 0.00 |
| Isolation | 618.67 | 191.46 | 0.00 |
