# Supplementary material for: Geographical and social isolation drive the evolution of Austronesian languages
Source: PLoS One. 2020 Dec 1;15(12):e0243171. doi: 10.1371/journal.pone.0243171 (PMC7707576; doi:10.1371/journal.pone.0243171)
Supplement: S5 Table — (DOCX) [file pone.0243171.s007.docx]

**Table S5.** Word gains, losses and overall lexical differences between the languages in each sister pair.

| Pair Number | Pair A | Pair B | Gain A | | Gain B | | Diff gains | | Loss A | | Loss B | Diff Loss | Total A | Total B | Diff Total |
| --- | --- | --- | --- | --- | --- | --- | --- | --- | --- | --- | --- | --- | --- | --- | --- |
| 1 | Tsou | CiuliAtayal | | 120 | | 68 | | 52 | | 106 | 53 | 53 | 226 | 121 | 105 |
| 2 | Bunun | CentralAmis | | 132 | | 177 | | -45 | | 45 | 34 | 11 | 177 | 211 | -34 |
| 3 | SubanunSindangan | Binukid | | 50 | | 31 | | 19 | | 76 | 61 | 15 | 126 | 92 | 34 |
| 4 | IsnegDibagat | Gaddang | | 38 | | 54 | | -16 | | 51 | 44 | 7 | 69 | 98 | -29 |
| 5 | IfugaoAmganad | BontokGuinaang | | 36 | | 56 | | -20 | | 65 | 60 | 5 | 101 | 116 | -15 |
| 6 | DayakNgaju | MerinaMalagasy | | 68 | | 135 | | -67 | | 38 | 138 | -100 | 106 | 273 | -167 |
| 7 | KelabitBario | KayanUmaJuman | | 76 | | 66 | | 10 | | 54 | 59 | -5 | 130 | 125 | 5 |
| 8 | Iban | Melayu | | 43 | | 27 | | 16 | | 51 | 57 | -6 | 94 | 84 | 10 |
| 9 | Chamorro | Palauan | | 138 | | 143 | | -5 | | 21 | 47 | -26 | 159 | 190 | -31 |
| 10 | Sika | Kedang | | 99 | | 107 | | -8 | | 41 | 49 | -8 | 140 | 156 | -16 |
| 11 | ESKamberaSouthern | LamboyaLaboya | | 32 | | 40 | | -8 | | 83 | 114 | -31 | 115 | 154 | -39 |
| 12 | RotiTermanu | Atoni | | 97 | | 124 | | -27 | | 18 | 46 | -28 | 115 | 170 | -55 |
| 13 | Numfor | Waropen | | 137 | | 184 | | -47 | | 38 | 33 | 5 | 175 | 217 | -42 |
| 14 | Manam | Wogeo | | 94 | | 87 | | 7 | | 36 | 34 | 2 | 130 | 121 | 9 |
| 15 | Motu | Mekeo | | 94 | | 134 | | -40 | | 24 | 63 | -39 | 118 | 197 | -79 |
| 16 | Bwaidoga | Dobuan | | 70 | | 66 | | 4 | | 66 | 65 | 1 | 136 | 131 | 5 |
| 17 | Chuukese | Woleai | | 42 | | 29 | | 13 | | 31 | 23 | 8 | 73 | 52 | 21 |
| 18 | Tongan | Samoan | | 33 | | 35 | | -2 | | 50 | 52 | -2 | 83 | 87 | -4 |
| 19 | FutunaEast | Niue | | 12 | | 15 | | -3 | | 43 | 86 | -43 | 55 | 101 | -46 |
| 20 | Rennellese | FutunaWest | | 11 | | 3 | | 8 | | 29 | 43 | -14 | 40 | 46 | -6 |
| 21 | Kapingamarangi | Luangiua | | 5 | | 4 | | 1 | | 28 | 29 | -1 | 33 | 33 | 0 |
| 22 | Maori | Hawaiian | | 32 | | 38 | | -6 | | 56 | 48 | 8 | 88 | 86 | 2 |
| 23 | Dehu | Nengone | | 190 | | 185 | | 5 | | 22 | 30 | -8 | 212 | 215 | -3 |
| 24 | SyeErromangan | Kwamera | | 57 | | 57 | | 0 | | 103 | 94 | 9 | 160 | 151 | 9 |
| 25 | Nahavaq | Mota | | 36 | | 81 | | -45 | | 99 | 106 | -7 | 135 | 187 | -52 |
| 26 | KwaraaeSolomonIslands | Toambaita | | 43 | | 37 | | 6 | | 33 | 49 | -16 | 76 | 96 | -20 |
| 27 | Simbo | Roviana | | 138 | | 82 | | 56 | | 75 | 25 | 50 | 213 | 107 | 106 |
