# Supplementary material for: Geographical and social isolation drive the evolution of Austronesian languages
Source: PLoS One. 2020 Dec 1;15(12):e0243171. doi: 10.1371/journal.pone.0243171 (PMC7707576; doi:10.1371/journal.pone.0243171)
Supplement: S6 Table — (DOCX) [file pone.0243171.s008.docx]

**Table S6.** GVIF values for each variable in the full models predicting word gains (top), word losses (middle) and overall lexical turnover (bottom)

| **Model** | Population size | Geographical isolation | Conflict within communities | Conflict with same culture | Conflict with other cultures |
| --- | --- | --- | --- | --- | --- |
| **Word gains** | 1.16 | 1.29 | 1.25 | 1.24 | 1.12 |
| **Word losses** | 1.33 | 1.17 | 1.33 | 1.41 | 1.26 |
| **Lexical turnover** | 1.33 | 1.20 | 1.09 | 1.19 | 1.17 |
