# Supplementary material for: Geographical and social isolation drive the evolution of Austronesian languages
Source: PLoS One. 2020 Dec 1;15(12):e0243171. doi: 10.1371/journal.pone.0243171 (PMC7707576; doi:10.1371/journal.pone.0243171)
Supplement: Attachment — Submitted filename: Response to reviewers.docx [file pone.0243171.s014.docx]

Dear Dr Calafell

Thank you very much for giving us the opportunity to further revise our manuscript. We now hope to have addressed all the additional questions raised by Reviewer 2 and hope that you are satisfied to with the result.

Below we specify how we have modified the Text and Supplementary Materials according to the specific comments by the reviewer.

*Re. In the end of the first paragraph you write: “Nonetheless, given a vast corpus of research has emerged on the evolutionary, ecological and social correlates of the global distribution of linguistic diversity.” Should the word “given” be deleted from the sentence? It would make more sense to me that way.*

Thank you very much for spotting this. It has now been corrected.

*Re. To me it sounds that in the Thomason example the point in changing the language is not so much in cultural group marking but instead in making the language mutually unintelligible for warfare reasons: “-- so that the enemies wouldn’t understand them”. Would the intentional changes in the language of Delaware Indians apply only in situations where they are in contact with Iroquois? Please clarify whether it is more about mutual unintelligibility or cultural group marking.*

There are a whole suit of examples along the same lines in the cited reference of Thomason (2007) where she explicitly relates this “cultural group marking” use of language with deliberate changes in grammar and phonology. Warfare is an extreme example of the phenomenon, where cultural groups with similar languages (otherwise changes would not be necessary) modify them so as to preserve inter-group but reduce inter-group intelligibility. The idea here is just like in the Nettle and Dunbar 1997 model – in situations of conflict, speakers of opposing sides (particularly when speaking related languages) will deliberately plan to modify their language fast enough so that enemies won’t be able to speak or understand their language, and by doing so, be able to pretend to be allies. The result is of course more differentiated languages, and thus stronger cultural markers.

*Re. Arelated --> a related*

This has been amended.

*Re. It would be clearer to present the study area “Here we provide the first integrated test of the effect of various sociodemographic and geographic features on linguistic diversification among 50 Austronesian languages.” before mentioning the problems of in using contemporary speaker populations as the data. In the current version the study area is first mentioned as an example case where using contemporary populations as the data is problematic.*

We thank the reviewer for the suggestion. However, we believe it makes more sense to introduce the question we are addressing first, and then our approach and dataset. This is why both in the abstract and main text we first make an effort to show that the puzzle of linguistic differentiation that the factors driving it remain unsolved. This is in fact the overall justification or our study; the choice of data is a subsequent step, which as mentioned by the reviewer, was affected by issues regarding data/population suitability.

We tried to think of a way of introducing the information about our case study earlier, but we believe that this would break our line of argumentation. However, we mention Austronesian languages in the title and on line 5 of the abstract, which we believe makes our case study and area clear to readers from the very beginning.

*Re. In table S1 there are 27 sister pairs listed even though based on the text there should be only 25 of them. In addition, it would be good to add to the Table S1 or to the Figure 1 which pairs belong to which of the 10 major subgroups of the Austronesian family (listed in the text) as this is not clear to a reader who is not familiar with the family. Please fix these.*

The two pairs corresponding to the branch lengths that represent the whole tree have now been removed (and therefore Table S1 only contains 25 pairs). We have also added an extra column specifying the Austronesian subgroups (according to those in the tree by Gray et al. 2009) to which each of the languages belong to.

Thanks for spotting it.

*Re. “(ii) using only tip branches also avoids the problem of non-independence between ancestor and descendant lineages within the phylogeny, as each branch is likely to be more similar in many traits to its immediate neighbours than to more distantly related branches”. It is not necessarily clear to the reader how similarity of immediate neighbours avoids the problem of non-independence. This was clearly explained earlier in the text but it would be good if you could clarify also this bit here.*

In other words, the problem of non-independence due to relatedness of the branches. We have now added this to the paragraph, which now reads as:

The sister-pairs approach has two main advantages over whole tree phylogenetic methods that use every branch in a phylogeny as a datapoint in an analysis, namely: (i) using only the tips of the phylogeny avoids the need to infer less reliable ancestral states down the phylogeny, which is particularly important given that some Austronesian languages date back as far as 4,500 years ago; and (ii) using only tip branches also avoids the problem of non-independence between ancestor and descendant lineages within the phylogeny, as each branch is likely to be more similar in many traits to its immediate neighbours than to more distantly related branches simply due to relatedness [22].

*Re. In section Social, demographic and geographic variables you write “Data on population size, geographical isolation and conflict within and between cultures--“ It first seems that the list has only four variables instead of the five included in the study and the reader may think whether one of the variables were taken from some other source. However, if all of them were from this source but the two between-cultures variables were grouped together, it would be good to clarify which is the case.*

No variables were grouped together, and indeed all of them were taken from the same source. We have amended that sentence and hope to have made that clearer. It now reads as

All social, geographic and demographic data used for our analyses was obtained from the “traditional state” section in Pulotu database, which concerns the state of cultural groups prior to large-scale modernisation [82].

*Re. “This selection process resulted in 27 pairs (n=54 languages) of Austronesian languages.” To make sure the reader is not confused with the number of language pairs used in this study, it would be good to note here again why the final number of pairs was 25 with reference “see above”.*

This has now been done.

*Re. Table S6 was not included in the Supplementary Material. Please add this.*

Sorry about that. This has now been included.

*Re. “If a word form found in one sister language has a cognate in other languages in the language family, it is likely to have been inherited from the common ancestor. This implies that the absence of that cognate form in the other sister language must be due to its loss after divergence from their exclusive common ancestor.” Would you please clarify is the whole phylogeny or only the sub-branch in question taken into account when checking the commonness of a certain cognate when counting the rates of word gains and losses? It sounds like a quite a rough requirement if the cognate form needs to be found from all the other languages of the family except from one of the two sister branches. Could you elaborate this?*

Thanks. We clarify again that the classification of cognates (in other words, the 210 basic semantic units) was not done for the present study but were taken from a peer-reviewed, established database (the Austronesian Basic Vocabulary Database): <https://abvd.shh.mpg.de/austronesian/>, detailed in Greenhill et al. (2008). The authors of the database determined which words represented cognates across the whole phylogeny, as well as which words represented borrowings in each of the languages (and thus needed to be excluded from the cognate lists of each of the languages).

*Re. “We did not include any identified loan words in the analysis--“ Does this mean that some of the 210 basic semantic units were excluded from some sister pair comparisons because there were borrowings? What was the source of information about the existence of borrowings? It would be good to clarify this so the reader knows e.g. how large part of the data was excluded due to the existence of loanwords.*

Please see the comment above.

*Re. “The total number of gains, losses, and non-informative results--“ Please elaborate what are these “non-informative results”. Please also add to this section how the lexical turnover was calculated.*

This has been modified accordingly, and we have moved the description of the lexical turnover variable from the statistical analysis section to this paragraph. Now it reads as

The total number of gains and losses were counted for all available semantic categories for each pair of languages using the Python package RateCounter developed by Simon Greenhill (https://github.com/SimonGreenhill/RateCounter; see [22] and Table S5). The overall rate of lexical turnover was then computed for each language pair by adding the number of gains and losses.

*Re. Parethesis is missing after the list of five predictors in the second paragraph of Statistical analysis section.*

Thank you for your attention, we have added the parenthesis.

Discussion
*Re. You say in the introduction that “Here we provide the first integrated test of the effect of various sociodemographic and geographic features on linguistic diversification--“. However, as none of these variables acts in isolation (e.g. some isolated groups are small some large, some small isolated groups have a lot of conflict with other cultures while some may have only little), it would be very relevant to study and discuss also the interactions between the studied variables. For example, you write “We found that being geographically isolated (--) greatly speeded up the rate of word losses and also moderately that of word gains. This is consistent with the idea that geographical isolation results in a higher risk of random loss of cultural items due to incomplete inter-generational sampling of existing variation” Does this inference of incomplete inter-generational sampling apply for both small and large populations? This is important especially as in the discussion in the population size paragraph you write “--smaller populations should be more prone word losses due to random sampling effects [16,17,19,23]--”*

This is a relevant question. The fact that our results show that both population size and geographical isolation exert a positive effect on the rate of word loss alongside the fact that those two variables are not correlated, indeed indicate that *ceteris paribus,* more isolated languages will lose terms faster (regardless of their speaker population size) and that languages with less speakers (regardless of how geographically isolated they are) will lose terms faster. However, whether the two variables together generate a positive feedback (i.e. interact) affecting the *magnitude of the speed* at which this happens is a question that would require a different analysis, as with the sister-pairs approach we cannot estimate rates of change of single languages but relative rates of change of sister-pairs compared to each other.

Although we believe that potentially all factors could interact with one another~~,~~ such analysis goes beyond the scope of the present study due to a lack of both methodology and data. Here, we wanted to test the simplest possible model, that is, whether each of those variables exerted independent, additive effects on rates of lexical change. We thank the reviewer for the suggestion, which we may address in future studies.

*Re. “In summary we argue that contact has contributed to differentiation among Austronesian languages mostly by preventing the random loss of existing lexical items, despite also hindering the ability of novel lexical innovations to emerge and be utilised”. I don’t quite see why you argue that contact has contributed to the differentiation if you say that contact both prevents the random loss of lexical items and hinders novel lexical innovations. To me this sounds that when contact takes place, differentiation does not happen. Please clarify this.*

Thanks for pointing this out. This was a mistake. We wanted to say geographical isolation (or lack of contact) instead of contact here. We replaced it in the text and the sentence makes sense now:

In summary, we argue that geographical isolation may have contributed to differentiation among Austronesian languages mostly by preventing the random loss of existing lexical items, despite also hindering the ability of novel lexical innovations to emerge and be utilised [38,59], rather than by affecting the internal social dynamics of isolated groups

*Re. When using only historical population size as predictor, your results for word gains were: estimate= 0.09, 90% HPDI: [0.01, 0.18] and for word losses: estimate = 0.01, 90% HPDI: [-0.10, 0.12]. When using the contemporary population size the results obtained with your method were for word gains: 0.06, 90% HPDI: [-0.06, 0.17] and for word losses: = 0.01, 90% HPDI: [-0.10, 0.12]. The result for word loss is exactly the same suggesting that the usage of historical vs. contemporary population size data does explain the difference between your and Greenhill et al’s results concerning word losses. In addition, the difference in the results of word gains also looks very minimal even though it changes a significant result obtained with the historical population size to non-significant when using contemporary population size. These issues should be taken into account when discussing the possible reasons why your results differ from those of Greenhill et al’s.*

Again, sorry about that and thank you for pointing it out. We had made a typo which has now been corrected. However, in effect discrepancies cannot be attributed to choice of statistical methods. We think that it is a combination of a) only including population size and b) the choice of the variable.

*Re. Related to the previous comment, it would be good to mention how much smaller your data were compared to Greenhill et al’s*.

The studies by Bromham et al. 2015 and Greenhill et al. 2010 included 10 and 81 language pairs respectively. We have now added this to the main text.

*Re. I believe reference to Fig 2 in the end of the fifth paragraph of Discussion should be Fig 1.*

Indeed. Thank you for pointing that out.

We would like to thank you very much again and for the very detailed and useful comments on our manuscript.

Best,

Cecilia Padilla-Iglesias, Erik Gjesfjeld and Lucio Vinicius
